# Isolation and Purification of Protamine from the Cultured *Takifugu flavidus* and Its Physicochemical Properties

**DOI:** 10.3390/molecules29010263

**Published:** 2024-01-04

**Authors:** Shuji Liu, Yue Zhang, Yihui Chen, Yongchang Su, Bei Chen, Yin Wang, Min Xu, Kun Qiao, Shuigen Li, Zhiyu Liu

**Affiliations:** 1 Key Laboratory of Cultivation and High-Value Utilization of Marine Organisms in Fujian Province, Fisheries Research Institute of Fujian, National Research and Development Center for Marine Fish Processing (Xiamen), Xiamen 361013, China; cute506636@163.com (S.L.); suyongchang@stu.hqu.edu.cn (Y.S.); chenbeifjfri@foxmail.com (B.C.); wangyin_83@163.com (Y.W.); xumin1315@foxmail.com (M.X.); qiaokun@xmu.edu.cn (K.Q.); 2 College of Food Science, Fujian Agriculture and Forestry University, Fuzhou 350002, China; zebra_99@126.com (Y.Z.); harris2197395@163.com (Y.C.); 3 Fujian Fisheries Technical Extension Station, Fuzhou 350002, China

**Keywords:** cultured pufferfish, protamine, purification, properties

## Abstract

Protamine is a cationic peptide derived from fish sperm and has several important functional properties: antibacterial properties, acting as a carrier for injectable insulin and as a heparin antagonist, combatting fatigue, etc. Thus, it has been widely used in medicinal applications and food products. Cultured *Takifugu flavidus* is a type of pufferfish with a delicious taste that is popular in China, and its production is increasing significantly. Therefore, protamine was extracted via acid extraction from the sperm of *Takifugu flavidus* and further isolated and purified via sephadex gel chromatography, ion exchange chromatography, and desalination chromatography. Furthermore, the physicochemical properties of protamine were investigated. The results showed that the sperm of the cultured *T. flavidus* were non-toxic, and the extracted and purified protamine had high contents of arginine (36.90%) and lysine (27.02%), respectively. The secondary structure of protamine was mainly β-folded and irregularly curled. Additionally, protamine exhibited high thermal stability with a denaturation temperature of 176 °C. This study would provide a theoretical basis for the structural analysis, bioactivity, and resource development of pufferfish protamine and help to promote the development of the pufferfish industry.

## 1. Introduction

Pufferfish, also known as bubble fish, blowfish, and fugu, belongs to the class Actinopterygii, order Tetraodontiformes, and family Tetraodontidae [1], and it is a popular culinary treat in Asia. The wild pufferfish contain tetrodotoxin with strong toxins, and the accumulation of toxin could be prevented after the pufferfish is cultured in captivity. Currently, several toxin-free species of pufferfish are cultured in China, including *Takifugu flavidus*, *Takifugu obsurus*, *Takifugu bimaculatus,* and *Takifugu rubripes* [2]. Among them, *Takifugu flavidus* has tender flesh, rich nutritional composition, and delicious taste, resulting in its extensive farming in the Fujian province. According to the Fisheries Yearbook of China [3], the aquaculture production of pufferfish in Fujian reached 10,185 tons in 2022, accounting for 61.3% of the national aquaculture production of pufferfish. Since the release of regulations by the Ministry of Agriculture and the State Administration for Market Regulation in 2016, the farming of pufferfish is only allowed for the consumption of the skin and flesh, and processing can only take place in registered pufferfish processing plants [2]. As a result, a significant amount of by-products are generated each year due to pufferfish farming [4]. The sperm contain abundant nutrients, accounting for approximately 7% of the fish’s body weight, and they have been confirmed to be non-toxic [5]. However, these sperm are discarded as waste, leading to substantial resource wastage.

Protamine is a highly cationic peptide and has been isolated from the sperm of a number of vertebrates and copepods, and it could replace the histone in sperm cell nuclei during sperm maturation [6]. Protamine can compress DNA into tight complexes; thus, it has at least three common applications. In clinical applications, protamine has been used as a carrier and slow-release agent for injectable insulin for decades. Moreover, the antibacterial properties of protamine are active against Gram-positive and Gram-negative bacteria, leading to applications in food preservatives [7]. The sperm of cultivated pufferfish contain a type of alkaline protein with a high application value named protamine. Protamine is a natural polycationic peptide that is rich in arginine and exhibits temperature resistance. It can be classified as monomer protamine, dimer protamine, and trimer protamine according to the different numbers of alkaline amino acids. It is considered to be a high-quality protein resource with significant nutritional and functional properties [8], which means that it has a wide range of potential applications: lowering blood pressure, inhibiting tumors, combating fatigue, strengthening liver function, producing broad-spectrum antibacterial effects, neutralizing heparin, etc. Therefore, protamine, as a novel natural food preservative, is suitable for the processing and storage of alkaline and thermosensitive foods. Apart from its promising applications in the food industry, protamine also plays an irreplaceable role in the medical clinical field; it is currently the only effective anticoagulant in extracorporeal circulation heart surgeries, antagonizing heparin. Nowadays, most current research studies on protamine mainly focus on extraction processes, antimicrobial activities, and the neutralization of heparin [9], which is primarily derived from salmon [10], trout [11], herring [6], and other fish species. The most comprehensive review of the structure and properties of protamine was published in 1973 [9]. The work mentioned above provides the most complete description of the field to date. Although most of the original publications described rather complicated flow schemes, limited research has been conducted on the structural and functional properties of protamine.

Research has revealed variations in both the content and composition of protamines among different fish species, with discrepancies even observed within the same species due to variations in the maturity of fish gonads. The aquaculture production of *T. flavdidus* has been steadily increasing in Fujian Province. In order to fully utilize the gonad resources of *T. flavdidus*, this paper primarily focuses on protamine extracted from cultivated *T. flavdidus.* Various chromatographic techniques, including molecular sieving, ion exchange, and desalting, are employed for separation and purification [12]. The relative molecular weight, secondary structure, and thermal denaturation properties of fish protamine are characterized using amino acid composition analysis, Fourier transform infrared spectroscopy (FTIR), circular dichroism (CD), and differential scanning calorimetry (DSC). The aim of this study is to elucidate the structural characteristics of protamine obtained from cultivated pufferfish, and it provides a reference for the value-added utilization of pufferfish sperm in aquaculture.

## 2. Results and Discussion

### 2.1. Toxicity and Nutritional Analysis of Cultivated Pufferfish Sperm

#### 2.1.1. Toxicity Analysis of Cultivated Pufferfish Sperm

Tetrodotoxin (TTX) is a naturally highly toxic non-protein neurotoxin found in most wild pufferfish [13]. According to the concentration of tetrodotoxin, TTX can be classified into different levels of toxicity. When the level of TTX is lower than 2.2 µg/g, it is considered non-toxic (D level); when TTX ranges from 2.2 µg/g to less than 22 µg/g (a single ingestion of 100 g is not toxic), it is classified as attenuated (C level); when TTX ranges from 22 µg/g to less than 220 µg/g (a single ingestion of less than 10 g will not cause toxicity), it is classified as highly toxic (B level); when TTX ≥ 220 µg/g (single ingestion of 1 g can be lethal), it is considered as extremely toxic (A level) [14]. The toxicity of cultured pufferfish is reduced due to changes in the food chain and environment [15]. Currently, the main species of cultured pufferfish include *Takifugu rubripes*, *Takifugu obscurus*, *Takifugu flavidus*, and *Takifugu porphyreus*. Therefore, the tetrodotoxins contained in the sperm of these four species of pufferfish were monitored, and the results are shown in Table 1. From the table, tetrodotoxin was not detected in the sperm of the four cultured pufferfish, and the levels were below the non-toxic limit (2.20 µg/g), belonging to the D level, which indicated that the sperm of the four cultured pufferfish were safe and non-toxic, and this is consistent with the research results of Ikeda [16], Shiro Itoi [17], etc.

#### 2.1.2. Nutritional Composition Analysis of the Sperm of Pufferfish

The protein, amino acid, mineral, and other nutritional components of the cultured pufferfish were tested, and the results are shown in Table 2. The crude protein content of the sperm of cultured pufferfish was 17.47 ± 0.49%, which was higher than the protein content of cultured *T. obscurus* (15.97–17.15 g/100 g) measured by Wang Liya et al. [5]. This difference may be attributed to factors such as pufferfish species, feeding methods, farming environment, and fishing season. The moisture content was 79.01 g/100 g, which was higher than that of bonito sperm (76.36%) [18]. The crude fat content in cultured *T. flavidus*’s sperm was (1.71 ± 0.12) g/100 g, which was higher than the crude fat content in its flesh and skin [19]. According to the “Management Regulations for Food Nutrition Labels,” the protein content of cultured *T. flavidus* in the sperm was ≥12 g/100 g (solid), and the fat content was ≤3 g/100 g (solid) [20]. Therefore, it can be explained that the sperm of pufferfish have the characteristics of high protein and low fat content. 

Among the mineral elements, the content of potassium was the highest, followed by phosphorus and calcium, which were the lowest. The zinc content of trace elements was (4.67 ± 0.21) mg/kg, which was much higher than that of sturgeon sperm (2.20 mg/kg) [21]. This made the sperm of pufferfish a rich source of zinc, which was easily absorbed and utilized by individuals with zinc deficiency. It was also beneficial for enhancing immune function and improving sexual function in the human body [22]. The selenium content was relatively low at only (0.18 ± 0.06) mg/kg.

The sperm of cultured *T. flavidus* contained 17 kinds of amino acids, with a higher proportion of basic amino acids and umami amino acids, among which the content of basic amino acids (Arg and Lys) accounted for 22.71% of the total amino acids because the sperm contained a kind of alkaline protein: protamine. Lysine, which can relieve emotional anxiety and enhance collective immunity, was mainly found in animal-based foods and legumes. Consuming the sperm of farmed pufferfish can help supplement the deficiency of lysine in cereal-based foods [23]. As a precursor of many biological compounds in mammals, arginine can participate in the ornithine cycle in the human body, reducing blood ammonia levels [24]. It can also promote the body’s production of sperm, provide energy for sperm, and enhance sexual function. The content of umami amino acids (Glu, Asp, Ala, and Gly) in the sperm of cultured pufferfish accounted for 38.33% of total amino acids. The content of umami amino acids was higher than that in pufferfish flesh (35.88%–38.71%), tuna sperm (12.71%) [25], western abalone (23.00%), and Peruvian squid (21.59%) [26], which could be the reason why people in ancient times enjoyed eating the sperm of Fugu and gave them the nickname “Xishi Milk,” referring to their delicious taste.

The content and composition characteristics of essential amino acids (EAAs) in the human body are the key factors for evaluating the nutritional value of proteins [27]. The ratio of ∑EAA/∑NEAA in the sperm of cultured pufferfish was 60.3%, which was close to the ideal amino acid composition of high-quality proteins proposed by FAO/WHO [28], indicating that the protein quality of pufferfish sperm was better, and the amino acid types were complete. Furthermore, the amino acid score (*AAS*), chemical score (*CS*), and essential amino acid score (*EAAI*) were calculated according to the FAO/WHO amino acid scoring pattern and egg protein scoring pattern (Table 3). Lysine had the highest score, while methionine + cystine was the lowest, suggesting that methionine + cysteine was the first limiting amino acid, and pufferfish gonads are rich in lysine but deficient in cysteine and methionine. Lysine is one of the essential amino acids in the human body, and it is the first limiting amino acid in cereal-based foods [29]. Consuming pufferfish sperm can help supplement lysine. Wu Yingying et al. [30] observed that methionine + cysteine had the highest content (115.86~126.30 mg/gprot) in the amino acid composition of *Flammulina velutipes*. If these two amino acids are consumed together, they could improve the utilization of proteins in the body. The *EAAI* value is an indicator for evaluating the nutritional value of dietary proteins based on the essential amino acids of whole egg protein [31]. According to the *EAAI* value, proteins can be classified into four categories: high-quality protein source (*EAAI* > 0.95), good protein source (0.86 < *EAAI* ≤ 0.95), usable protein source (0.75 ≤ *EAAI* ≤ 0.86), and inadequate protein source (*EAAI* < 0.75) [32]. The *EAAI* value of cultured pufferfish sperm was only 0.27, indicating that the amino acid nutritional value of pufferfish sperm was relatively low, and it is unsuitable for being the main protein source in diets.

### 2.2. Isolation and Purification of Crude Protamine from Cultured T. flavidus

The protamine of *T. flavidus* was extracted via acid extraction and alcohol precipitation. The yield rate of the extraction was 18.87%, which was higher than the extraction rate of 3.82% with respect to *Takifugu rubripes, as* reported by Jiayuan Z et al. [33]. This difference may be attributed to the moisture content and varieties of species of pufferfish used [34]. The crude protamine of pufferfish was further purified via glucose gel filtration chromatography (Sephadex G50), carboxymethyl agarose gel FF ion exchange chromatography (CM Sepharose Fast Flow, CMFF), and desalting chromatography (Sephadex G25 Fine). The identification and determination of the relative molecular weight of protamine were carried out using the Sakaguchi reaction [35].

#### 2.2.1. UV Absorption Spectrum of Protamine of *T. flavidus*

**Figure 1:**
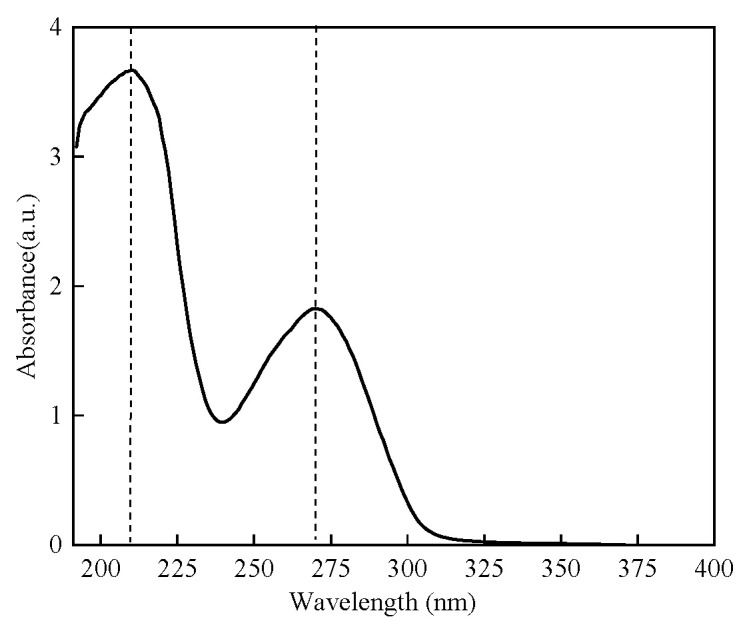
The UV absorption spectrum of protamine from *T. flavidus*.

Crude protamine was extracted from the sperm of *T. flavidus* via a UV absorption spectrum, and the results are shown in Figure 1. The spectrum revealed two absorption peaks around 215 nm and 270 nm, with the peak near 215 nm being the highest: this could be attributed to the stronger ultraviolet absorption of peptide bonds in crude proteins compared to aromatic amino acids [36]. Additionally, the crude protamine contained a significant amount of phenylalanine and tyrosine (as shown in Table 2), both of which had conjugated double bonds and exhibited absorption properties within the ultraviolet range [37]. Therefore, a detection wavelength of 215 nm would be chosen for subsequent purification experiments.

#### 2.2.2. Glucose Gel Column Chromatography Profile of Protamine from *T. flavidus*

**Figure 2:**
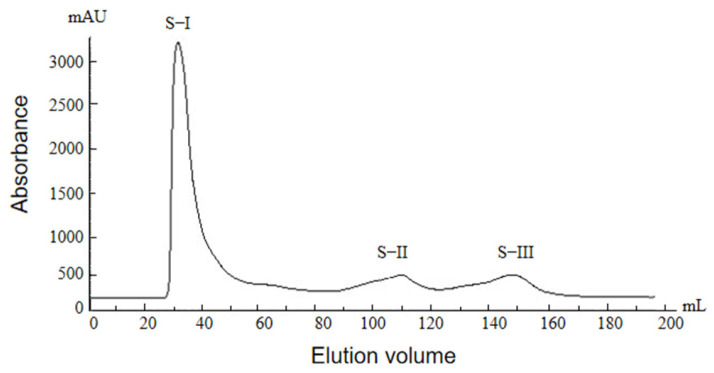
Glucose gel chromatography of protamine from *T. flavidus*.

The components of crude protamine were complex, and the relative molecular weight was greater than thousands of Daltons [35]. Sephadex G50 gel column chromatography with a detection wavelength of 215 nm was employed for the separation and purification of the extracted crude protamine. The results are shown in Figure 2. Three peaks (S-I, S-II, and S-III) were observed in the crude protamine of *T. flavidus* via sephadex G50 gel chromatography. The Sakaguchi reaction was implemented, and an antibacterial activity assay was carried out with respect to these three peaks. It was observed that the peak S-I displayed a red color in the Sakaguchi reaction, indicating its antibacterial activity. On the other hand, the peaks of S-II and S-III did not change color relative to the Sakaguchi reaction and exhibited no antibacterial activity (Table 4). Therefore, the peak S-I was selected as the target peak and collected, desalted, and freeze-dried for further separation and purification.

#### 2.2.3. Ion Exchange Chromatography Profile of Protamine from *T. flavidus*

**Figure 3:**
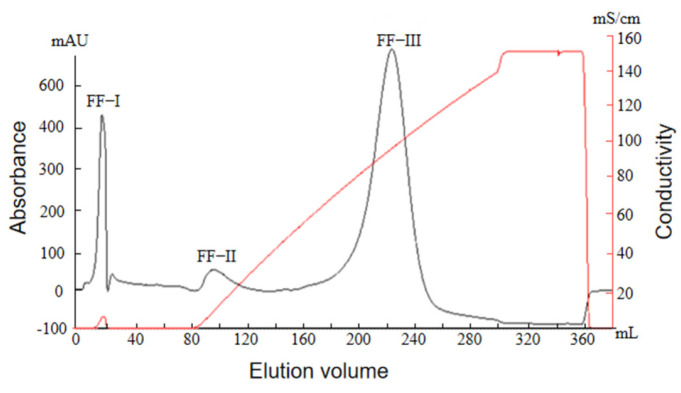
CM Sepharose Fast Flow ion exchange chromatography of protamine from *T. flavidus*.

Protamine is essentially a kind of alkaline polycationic peptide. Therefore, the peak S-I purified via sephadex G50 gel chromatography was further purified via cation-exchange chromatography–hydroxymethyl agarose gel FF ion exchange chromatography (CM Sepharose Fast Flow); the results are shown in Figure 3. From the graph, it can be observed that three peaks, namely FF-I, FF-II, and FF-III, are separated via linear elution with a 0.2–1.8 mol/L NaCl buffer after separation via hydroxymethyl agarose gel chromatography. Because the peak FF-I is observed at 0~80 mL, this indicates that it was not adsorbed in the column, and it is considered a penetration peak. While the Sakaguchi reaction and antibacterial activity of peaks FF-II and FF-III were tested, respectively, it was observed that peak FF-II exhibited a weak Sakaguchi reaction, but no antibacterial activity and peak F-III exhibited a significant Sakaguchi reaction and good antibacterial activity (as shown in Table 4). Therefore, peak FF-III was selected as the target protein peak for collection.

#### 2.2.4. Desalination Column Chromatography Profile of Protamine from *T. flavidus*

**Figure 4:**
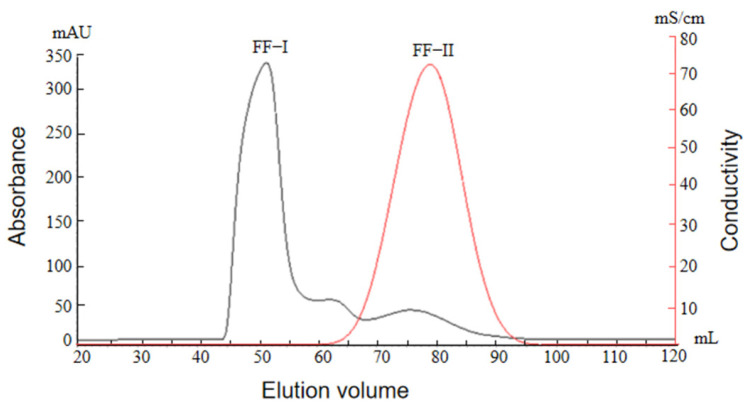
Desalination column chromatography of protamine from *T. flavidus*.

The target peak FF-III collected from CM Sepharose Fast Flow ion exchange chromatography using sephadex G50 gel exhibited high salt concentrations, requiring desalting before freeze-drying. Sephadex G25 is a well-performing gel filtration medium that is commonly used for desalination, the removal of small molecules, and buffer exchange in industrial applications [38]. Compared to dialysis, sephadex G25 chromatography offers short desalting cycles, higher efficiency, and remarkable effects [39]. Therefore, sephadex G25 chromatography was employed for the desalting of peak FF-III, as shown in Figure 4. From the graph, it can be observed that there were two peaks (G-I and G-II) after the peak FF-III’s passage through the desalting column, and the distance between peaks G-I and G-I was relatively wide, which can be well separated, indicating that sephadex G25 chromatography could effectively separate the salts from peak F-III and achieve the desalting effect. Peak G-I was collected and freeze-dried, yielding purified pufferfish protamine (PPP).

### 2.3. Physicochemical Properties of Protamine of Protamine from T. flavidus

#### 2.3.1. Analysis of the Amino Acid Components of Protamine from *T. flavidus*

The amino acid composition of the crude protamine (CPP) and pure protamine (PPP) of *T. flavidus* was analyzed, respectively, and the results are shown in Table 5. From Table 5, it can be observed that compared to CPP, PPP exhibited a significant increase in the content of arginine and lysine, a slight increase in the phenylalanine, cysteine, and histidine content, and a decrease in the content of other amino acids. The relative content of arginine and lysine after purification was 36.90% and 27.02%, respectively, accounting for more than half of the total amino acids. They were the major amino acids in the protamine of *T. flavidus*. The proportion of total alkaline amino acids increased from 33.90% to 65.45%, which was significantly higher than that of tuna (39.00%) [24] and *Aristichthys nobilis* (33.4%) [40]. In addition to arginine and lysine, histidine was also detected, indicating that the protamine of *T. flavidus* belonged to trimer protamine. Compared with *T. obscurus* [5], the amino acid composition of protamine in testicular *T. flavidus* was more abundant. Both had the highest proportion of arginine and similar content, exceeding that of squid (9.61%) [41] and tuna (25.86%) [42]. However, these contents were lower than those observed in salmon (66.30%) [43], sardine (70.11%) [44], and hagfish (88.39%). The proportion of arginine content in the protamine of pufferfish was approximately 1/3, which differed significantly compared to the statement made by Park et al. [45], who reported that protamine comprised 2/3 as arginine residues and one-third as other amino acids. This difference may be attributed to variations in fish species, feed composition, aquaculture practices, rearing environment, geographical location, and climatic conditions [45].

#### 2.3.2. Fourier Infrared Spectroscopy (FTIR) Analysis of Protamine

**Figure 5:**
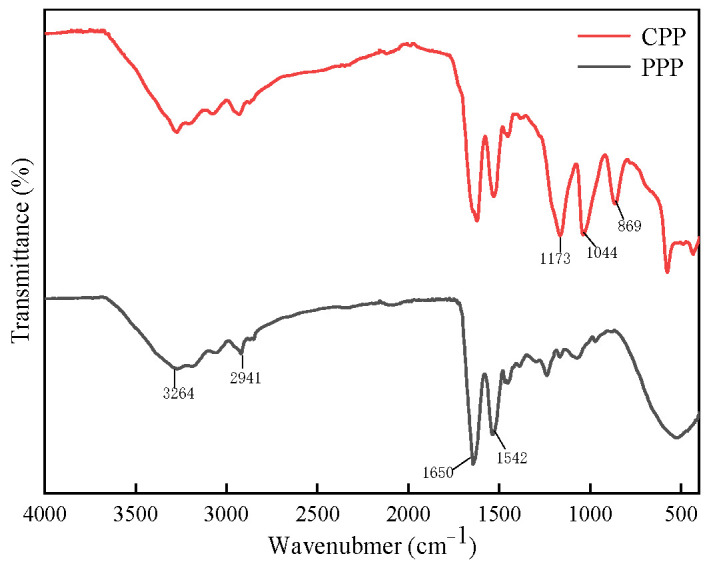
FTIR spectra of protamine from *T. flavidus*.

FTIR is a method that combines Fourier transform with infrared spectroscopy to detect subtle changes in protein structure. It has the following advantages: simple operation, small sample requirement, high sensitivity, good resolution, and a high signal-to-noise ratio [46]. Figure 5 shows the infrared spectra of the CPP and PPP of *T. flavidus*, in which the wavenumber position and intensities of strong and medium weak peaks collectively comprise the structural information of protamine. Moreover, both CPP and PPP exhibited similar absorption peaks with respect to multiple wavenumber ranges, exhibiting a high degree of consistency. Compared with the infrared spectra of CPP and PPP, the impurity interference peaks in PPP were significantly reduced, with noticeable changes occurring at 3 specific wavenumbers, namely 869, 1044, and 1173 cm^−1^. The peaks at 869 cm^−1^ and 1173 cm^−1^ correspond to polysaccharides, while the vicinity of 1044 cm^−1^ corresponds to the -OH group [47]. Considering the change in the amino acid content of *T. flavidus* protamine in Table 5 above, it could be inferred that the filtered part in the purification process might comprise some carbohydrate substances and amino acids with hydroxyl groups in the side chains, such as serine, threonine, and tyrosine. It was also possible that excessive residual ethanol was used in the extraction process, resulting in an increase in alcohols, esters, and other impurities in the samples [48].

The infrared spectra of protamine within the range of 1500~4000 cm^−1^ exhibited similar peak shapes to sturgeon, indicating the presence of characteristic peaks for protamine. Within the range of 3300~2700 cm^−1^, both CPP and PPP displayed broad and similar peaks, and they are primarily attributed to the stretching vibrations of CH bonds in the peptide chains and numerous hydrogen bonds between molecules or within molecules [49]. At 3264 cm^−1^, there was a peak corresponding to the amide A band, which represented the stretching vibration caused by the formation of a hydrogen bond association between -OH and -NH [50]. The absorption peak at 2941 cm^−1^ corresponded to the characteristic absorption of -CH_2_ [51]. Within the range of 1690~1500 cm^−1^, which is the region comprising double-bond stretching vibrations [52], both PPP and CPP exhibited a strong absorption peak at 1639 cm^−1^, and they were mainly affected by hydrogen bonding effects, resulting in a strong C=O stretching vibration peak near 1650 cm^−1^ under the solid-state condition, which belongs to the amide Ⅰ band (1700–1600 cm^−1^) [53]. It was situated between the β-sheet structure and the random coil region of the amide Ⅰ band, reflecting that the secondary structure of protamine consisted mainly of β-sheets and random coils [54]. At 1542 cm^−1^, there was a peak attributed to the overlapping absorption of C=N stretching vibrations and N-H bending vibrations, which belonged to the amide II band (1600–1500 cm^−1^) [55].

#### 2.3.3. Differential Scanning Calorimetry (DSC) and Thermal Analysis of Protamine

DSC is a method for the analysis of protein thermal stability that requires a small sample volume and offers high resolutions, which can aid in the determination of the thermodynamic effects of the conformational changes based on the heat-absorption and heat-release processes observed in the DSC curve. The negative peaks in the DSC curve corresponded to the endothermic denaturation of the substance. The higher the transition temperature and denaturation enthalpy value, the greater the thermal stability of the protein [56].

**Figure 6:**
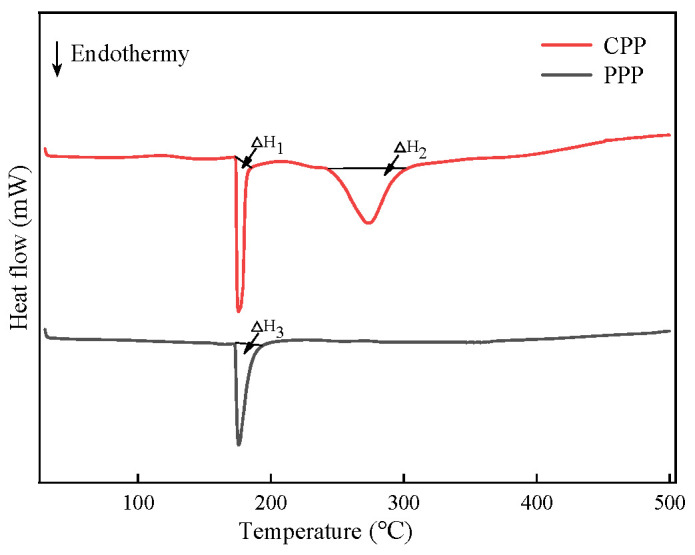
DSC absorption of protamine from *T. flavidus*.

Figure 6 shows the DSC of the CPP and PPP of *T. flavidus* within the temperature range of 40~500 °C. From the graph, it could be observed that CPP exhibited two denaturation peaks at 176 °C and 274 °C, with denaturation enthalpies of ΔH1: 73.92 J/g and ΔH2: 318.81 J/g, respectively, while PPP exhibited only one denaturation peak at 176 °C, with a denaturation enthalpy value of △H3:192.19 J/g. This indicated that the peak at 176 °C was the endothermic peak of the protamine of *T. flavidus*, while the peak at 274 °C was a result of impurities. The protamine of *T. flavidus* exhibited high denaturation temperatures, which were significantly higher than the denaturation temperatures of some common heat-resistant proteins, such as lactoferrin (70–75 °C) and whey protein (76–82 °C) [57]. It belonged to a highly thermally stable protein. Compared to CPP, the phase transition temperature of PPP did not change significantly, but the denaturation enthalpy value increased, indicating a lower number of impurities and improved thermal stability in PPP. There were many factors affecting the thermal stability of proteins, including amino acid composition, temperature, dipeptide composition, etc. [58]. The thermal stability of heat-resistant proteins was likely influenced by multiple factors. The high denaturation temperature of protamine may be attributed to its high content of arginine [35]. Arginine side chains contained fewer methyl groups and possessed multiple non-covalent bonds, such as ionic bonds and intermolecular forces [59]. The guanidine portion could reduce the activation energy of the chemical reactions and provide a larger surface area for charge interactions, resulting in arginine’s higher dissociation constant (pKa) and resonance stability [59]. It was also observed that heat-resistant proteins contained more hydrophobic and charged amino acids than mesophilic proteins [60]. According to the data in Table 5, the proportions of hydrophobic and charged amino acids in PPP were 30.34% and 65.45%, respectively, which may also contribute to the high thermal stability.

#### 2.3.4. Circular Dichroism (CD) Spectroscopy Analysis of Protamine

CD spectroscopy is one of the primary methods used to study the secondary structure of proteins, and it has been widely applied in protein conformation research. CD spectroscopy, particularly within the far-ultraviolet range (190–260 nm), can provide information about the arrangement of peptide bonds in proteins, allowing for the calculation of different secondary structures proportions, including α-helix, β-sheet, β-turn, and random coil [61]. By conducting far-ultraviolet CD spectroscopy on the protamine of *T. fluvidus*, the results presented in Table 6 show the distribution of secondary structures. According to Table 6, the β-sheet’s structure was the most abundant, accounting for 51.40% of the protamine, followed by the random coil (38.20%), while β-turn was present and exhibited the lowest proportion (10.40%). Moreover, the α-helix structure was almost undetectable, which may be related to the high content of alkaline amino acids (55.05%, in Table 5). Strong alkaline amino acids with similar charges tended to exhibit repulsive effects, preventing the formation of hydrogen bonds between amino acid residues and making the α-helix structure unstable and prone to disruption [62]. Therefore, according to the results of circular dichroism spectroscopy, the secondary structure of protamine from *T. flavdidus* predominantly comprises β-folds, complemented by irregular coils. This observation aligns with the findings from the Fourier transform infrared spectroscopy.

## 3. Materials and Methods

### 3.1. Reagents and Instruments

The sperm of cultivated *T. flavdidus* were purchased from Fujian Fugu Fresh Aquatic Products Co., LTD. (Zhangzhou, China). Nitric acid, hydrochloric acid, sulfuric acid, perchloric acid, sodium citrate, anhydrous ethanol, sodium hydroxide, sodium hypochlorite, acetic acid, sodium acetate, and Coomassie bright blue R-250 were purchased from China National Pharmaceutical Group Chemical Reagent Co., Ltd., (Shanghai, China). Arginine, glycine, and naphthol were purchased from Shanghai Aladdin Biochemical Technology Co., LTD. (Shanghai, China). The tetrodotoxin ELISA test kit was purchased from Shandong Meizheng Biotechnology Co., LTD. (Rizhao, China). The PAGE gel rapid preparation kit (12.5%) was purchased from Shanghai Yase Biomedicine Technology Co., LTD. (Shanghai, China). Sephadex G 50, CM Sepharose Fast Flow, and sephadex G25 fine were purchased from Situfan Biotechnology Co., LTD. (Hangzhou, China). 0.45 μm and 0.22 μm filter membranes were purchased from Pall Filter Co., LTD. (Beijing, China).

The FA-1004 Electronic analytical balance was purchased from Shanghai Shunyu Hengping Scientific Instrument Co., LTD. (Shanghai, China). The SCIENTZ-10 N vacuum freeze dryer was purchased from Ningbo Xinyi Biotechnology Co., LTD. (Nibo, China). An RO DI pure water purifier was purchased from Shanghai Hetai Instrument Factory (Shanghai, China). A 5810 R high-speed centrifuge was purchased from Eppendorf (Hamburg, Germany). The KDN-103 f Kjeldahl nitrogen analyzer was purchased from Shanghai Fiber Inspection Instrument Co., LTD. (Shanghai, China). An SZF-06 A fat analyzer was purchased from Shanghai Xinjia Electronics Co., LTD. (Shanghai, China). An ETHOS1 microwave digestion system was purchased from MILESTONE (Vancouver, BC, Canada). The LA8080 amino acid automatic analyzer was purchased from Hitachi LTD. (Tokyo, Japan). The 101-2 AB electric blast drying oven was purchased from Tianjin Teste Instrument Co., LTD. (Tianjin, China). The DRHH-S6 constant thermostatic water bath was purchased from Shanghai Shuangjie Experimental Equipment Co., LTD. (Shanghai, China). The C-MAGHS7 magnetic stirrer was purchased from Aika China Instrument Equipment Co., LTD. (Guangzhou, China). The UV-2450 UV-visible spectrophotometer was purchased from Shimadzu Corporation (Kyoto, Japan). The AKTApure protein purification system and F9-R fraction collector were purchased from Sitofan Biotechnology Co., LTD. (Hangzhou, China). The V700 PHOTO gel imaging system was purchased from Epson (China) Co., LTD. (Beijing, China). The TENSORII Fourier Transform Infrared Spectrometer was purchased from Bruker Corporation (Saarbrucken, Germany). The DSC3 differential scanning calorimeter was purchased from Mettler—Toledo International LTD. (Shanghai, China). The J-810 circular dichroism spectrometer was purchased from Jasco Corporation (Shanghai, China).

### 3.2. Methods

#### 3.2.1. Nutritional Component Analysis

##### Determination of the Tetrodotoxin (TTX)

The toxicity of pufferfish was determined according to the instruction manual of the tetrodotoxin ELISA test kit. The toxicity classification standards are as follows: TTX ≥ 220 μg/g is classified as highly toxic (Grade A); 22 μg/g ≤ TTX < 220 μg/g is classified as strongly toxic (Grade B); 2.2 μg/g ≤ TTX < 22 μg/g is classified as weakly toxic (Grade C); TTX < 2.2 μg/g is classified as non-toxic (Grade D) [63].

##### Determination of Essential Nutrients and Mineral Elements

The determination of moisture, ash, crude protein, and crude fat was conducted according to the following methods [64]: moisture: GB 5009.3–2016 [65] (thermogravimetric method); ash: GB 5009.4-2016 [66] (incineration method); crude protein: GB 5009.5-2016 [67] (Kjeldahl method); crude fat: GB 5009.6-2016 [68] (Soxhlet extraction method).

Sample preparation: A total of 1.00 g of sperm from cultivated *T. flavdidus* was accurately weighed and placed in a digestion tank. A total of 10 mL of nitric acid and 0.5 mL of perchloric acid were added. After microwave digestion using a microwave digestion system, the flask was placed on a heating plate until the solution in the flask tank was colorless, transparent, or slightly yellow. After cooling, the solution was transferred to a 50 mL volume bottle; then, the volume was adjusted to 50 mL with water and then mixed for use.

Mineral elements were determined as follows: calcium (Ca) was determined according to GB 5009.92-2016 [69]; potassium (K) and sodium (Na) were determined according to GB 5009.91-2017 [70]; magnesium (Mg) was determined according to GB 5009 241-2017 [71]; phosphorus (P) was determined according to GB 5009.87-2016 [72]; zinc (Zn) was determined according to GB 5009.14-2017 [73]; selenium (Se) was determined according to GB 5009.93-2017 [74].

##### Amino Acid Composition Determination and Evaluation

Sample pretreatment [75]: A total of 1.00 g of testis tissue was weighed and placed in a 35 mL hydrolysis tube containing 15 mL of 6 mol/L hydrochloric acid solution. The tubes were frozen at 20 °C for 5 min, vacuumed, and filled with nitrogen gas for protection. Then, it was sealed with an alcohol blowtorch and then hydrolyzed in a 110 °C electric blast incubator for 22 h. After that, they were removed and cooled to room temperature. The hydrolysate was transferred to a 50 mL volumetric bottle, and the hydrolytic tube was rinsed with a small amount of ultrapure water several times. The rinsing solution was transferred to the volumetric bottle and shaken well at a constant volume. A total of 1 mL of the aliquot was accurately used for concentration until dryness under the reduced pressure at 55 °C. Then, it was dissolved in 1 mL of sodium citrate buffer at a pH of 2.2 and passed through a 0.22 μm filter membrane. The filtrate was prepared for analysis.

The conditions for the automatic amino acid analyzer are as follows: chromatographic column, 4.6 mm × 150.0 mm, 5 μm ion exchange resin; detection wavelength, 440 nm and 570 nm; buffer solution flow rate, 0.40 mL/min; ninhydrin reagent flow rate, 0.30 mL/min; the temperature of the separation column, 53 °C; the temperature of the reaction column, 135 °C; the pressure of the column was 3~4 MPa; injection volume of sample, 20 μL.

The measured essential amino acids are converted into milligrams of amino acids per gram of protein [5]. The amino acid score (*AAS*), chemical score (*CS*), and essential amino acid index (*EAAI*) of cultivated pufferfish sperm are calculated according to the amino acid ratio coefficient method and the amino acid scoring pattern recommended by the Food and Agriculture Organization of the United Nations (FAO)/World Health Organization (WHO) (mg/g N) and the amino acid pattern for egg proteins was as proposed by the Institute of Nutrition and Food Hygiene, Chinese Academy of Preventive Medicine (mg/g N), respectively. The equations are as follows:

(1) $$AAS=\frac{CAM}{CAFW}$$

(2) $$CS=\frac{CAM}{CAE}$$

(3) $$EAAI=\sqrt[{n}]{\frac{100\;a}{aE}\times \frac{100\;b}{bE}\times \frac{100c}{cE}\times \cdots \times \frac{100\;f}{fE}}$$

In Equation (1), *CAM* represents the content of amino acids in the sample that will be measured, mg/g N; *CAFW* represents the content of the same amino acids with respect to the FAO/WHO amino acid scoring model, mg/g N.

In Equation (2), *CAE* represents the content of the same amino acids in the whole egg protein scoring model. 

In Equation (3), *a*, *b*, *c*, …, *f* represents the essential amino acid content of the test sample; *aE*, *bE*, *cE*, …, *fE*, represents the essential amino acid content of whole egg proteins.

#### 3.2.2. Extraction of Protamine

Referring to the method by Gill [6], the lyophilized powder of cultivated pufferfish sperm was mixed with a NaCl solution (0.14 mol/L, pH8.5) at a ratio of 10:1, stirred for 30 min by magnetic force, and then centrifuged for 20 min at 10,000 r/min and 25 °C. The supernatant was discarded. A 0.6 mol/L sulfuric acid solution was added in a volume that was four times that of the precipitate. The mixture was extracted at 45 °C for 2.4 h and then centrifuged at 25 °C and 6000 r/min for 15 min; the supernatant was then collected. Anhydrous ethanol at 4 times its original volume was added to the supernatant to precipitate the protein, and it was held at 4 °C overnight. Then, the mixture was centrifuged at 8000 r/min for 20 min to collect the precipitation and freeze-dried to form crude protamine (CPP). The CPP was packaged and stored at −20 °C for later use.

#### 3.2.3. Protamine Separated by Sephadex G50 Gel Filtration Chromatography

A modified version of the method proposed by Aida Karray et al. [76] was used. An appropriate amount of sephadex G50 was weighed and swelled in pure water at a ratio of 1:10. The swelling process included incubation at 20 °C and 90 °C for 3 h and 1 h, respectively. After washing with water 2–3 times and discarding the supernatant, the swollen sephadex G50 was packed into a column (Φ1.00 cm × 100.00 cm), and it settled naturally. The packed gel column was free of bubbles and layers and exhibited a uniform appearance. The detection wavelength was set to 215 nm, and the flow rate was maintained at 1 mL/min with a column pressure below 1 MPa. The column was equilibrated with a pH of 5.4 and a 25 mmol/L acetic acid–sodium acetate buffer using 2–3 column volumes (CVs). Once the baseline stabilized, the protamine solution was filtered using a 0.45 μm microporous membrane before injection, with a sample volume of 5 mL per run. Each fraction of 4 mL was collected, and the UV detection peaks were subjected to a Bradford assay. Fractions containing the same components were combined, concentrated, and stored at −80 °C for future use.

#### 3.2.4. Protamine Separated by Carboxymethyl Cellulose Gel FF Ion Exchange Chromatography

A modified version of the method proposed by Hsieh and Meng-Shun et al. [77] was used. Carboxymethyl cellulose gel with carboxymethyl as the charged functional group was used as the column’s packing material. The gel was washed 3–4 times with an appropriate amount of distilled water to remove any residual ethanol adsorbed on the gel. Subsequently, the gel was washed 3 times with a pH of 8.0 and a 50 mmol/L glycine–sodium hydroxide (Gly-NaOH) buffer. The washed gel was then packed into a chromatography column (Φ1.6 cm × 10.0 cm). The detection wavelength was set at 215 nm with a sensitivity of 0.5 A. The flow rate of the constant current pump was 3 mL/min.

The wavelength was 215 nm, the sensitivity was 0.5 A, and the flow rate of the constant-current pump was 3 mL/min. The column was equilibrated with a 50 mmol/L Gly-NaOH buffer solution, and this equilibration step was repeated for 2–3 column volumes (CVs) until a stable baseline was obtained. The protamine solution with a concentration of 20 mg/mL was injected with a sample volume of 5 mL per run at a flow rate of 5 mL/min. Elution was performed with a 3 CV wash using the Gly-NaOH buffer, followed by gradient elution with the Gly-NaOH buffer containing 0.2–1.8 mol/L NaCl for 10 CV. Fractions were automatically collected at a rate of 2 min/tube, and fractions containing the same components were combined after the Bradford assay identification.

#### 3.2.5. Protamine Separated by Sephadex G25 Fine Chromatography

A modified version of the method proposed by Rajapakse et al. [78] was used. A total of 15.0 g of sephadex G25 fine was weighed and processed. The processed gel was then packed into a column (Φ2.6 cm × 10.0 cm). The detection wavelength was set at 215 nm, and the flow rate was maintained at 5 mL/min. The column was equilibrated with a Gly-NaOH buffer for 5 column volumes (CVs) until a stable baseline was obtained. The sample was then injected with a sample volume of 10 mL per run at a flow rate of 10 mL/min. Desalting was performed using ultrapure water as the eluent for 3 CVs. Fractions were automatically collected at a rate of 3 min/tube, and collection was stopped when conductivity exceeded 1 mS/cm (i.e., each desalting fraction had a volume of 15 mL). The collected desalted solution was freeze-dried to obtain purified protamine. 

#### 3.2.6. Sakaguchi Reaction of Protamine

Following the method described by Tom A. Gill et al. [6], 1.5 mL centrifuge tubes were used. Reagents—10% NaOH solution, 0.2% α-naphthol solution, and sodium hypochlorite solution—were added into the sample successively at a ratio of 10:5:1:1, and they were thoroughly mixed. Simultaneously, a 0.1% histidine solution was prepared as the positive control. The same procedure was repeated for the control. A color change in the solution was observed. The color of the solution containing protamine changed from colorless to red.

#### 3.2.7. Detection of the Maximum Ultraviolet Absorption Wavelength of Protamine

According to the method of Qiaozhen Bao et al. [79], the maximum ultraviolet absorption wavelength of protamine in Takifugu was determined using UV spectrophotometry. A protamine solution with a concentration of 1 mg/mL was centrifuged and filtered through a 0.45 μm membrane. The filtrate was then transferred into a clean quartz cuvette. A UV-visible spectrophotometer was used to scan and analyze the solution within the range of 190 nm to 400 nm.

#### 3.2.8. Fourier Transform Infrared Spectroscopy (FTIR) Measurement of Protamine

A modified version of the method proposed by Sirison et al. [80] was used. The OPUS software was employed for signal inspection and peak position recording. The resolution was set at 4 cm^−1^, and the number of scans was set at 32. The scanning range was from 4000 cm^−1^ to 400 cm^−1^. Before sample analysis, a background single-channel calibration was performed to obtain the blank value. The sample was then applied, and the probe was pressed downward to ensure good contact with the powdered sample. After previewing and adjusting the spectrum, the scanning process was initiated.

#### 3.2.9. Differential Scanning Calorimetry (DSC) of Protamine

Following the method described by Ashley C et al. [81], a differential scanning calorimeter was used to perform a thermal analysis of the protamine. A precisely weighed sample measuring 5.0 mg was placed in a dried and clean aluminum crucible, compacted, and sealed. An empty aluminum crucible without the sample was used as a blank reference. The software and instrument were used sequentially, and when the temperature reached 30 °C, the aluminum crucible containing the sample was inserted. The temperature range was divided into four segments: 30–100 °C, 100–200 °C, 200–300 °C, and 300–400 °C. The heating rate was set at 10 °C/min, and the purge gas flow rate was set at 70 mL/min. A complete temperature curve was obtained, and the peak values were used to determine the denaturation temperature (Td, °C); the enthalpy value (ΔH, J/g) was then calculated, integrating the peak area.

#### 3.2.10. Circular Dichroism (CD) Spectroscopy of Protamine

CD spectroscopy was used to determine the secondary structure of the protamine in the ultraviolet region. Following a modified version of the method proposed by Lavaraj Devkota et al. [82], a 1 mm quartz cuvette was used. The cuvette was washed with ultrapure water and dried. The background and blank buffer solutions were collected first within the wavelength range of 180–260 nm. Then, the protamine solution with a concentration of 0.2 mg/mL was added to the cuvette, and far-ultraviolet (178~250 nm) scanning and data collection were performed at room temperature. The parameters were set as follows: wavelength step size of 1 nm, a response time of 0.5 s, scanning speed of 50 nm/min, and sample examination was repeated 3 times.

#### 3.2.11. Data Processing

The experimental data were analyzed using SPSS 25.0 software with a one-way analysis of variance (ANOVA). The data are presented as “mean ± standard deviation,” with lowercase letters indicating significant differences between different groups (*p* < 0.05). Excel 2019 and Origin 2021 software were used for the statistical organization, analysis, and visualization of experimental data. Each group was set up with 3 replicates, and the experiment was independently repeated 3 times.

## 4. Conclusions

CPP was prepared from cultured *T. flvidus* sperm using an acid extraction method. Subsequent purification steps involving sephadex G50, CMFF, and sephadex G25 were performed, and the target peaks were collected after the Sakakibara reaction and antimicrobial activity testing. The freeze-dried product obtained from these processes was PPP. The amino acid composition of protamine was determined. It was observed that the amino acid types of protamine were complete. In particular, the contents of arginine and lysine were high at 36.90% and 27.02%, respectively. The protamine contained arginine, lysine, and histidine, indicating that it belonged to the trimer protamine group. The proportion of alkaline amino acids accounted for 65.45% of the total amino acids. Differential scanning calorimetry (DSC) results showed that the denaturation temperature of *T. flavidus* protamine reached 176 °C, indicating high thermal stability. Both the FTIR and CD spectroscopy confirmed that the secondary structure of protamine consisted of the β-sheet and random coil structures. Further research is required to investigate the detailed structure, antimicrobial activity, and underlying mechanisms of protamine in order to obtain deeper insights into its biological properties.

## Figures and Tables

**Table 1:** Results of tetrodotoxin detection in the sperm of 4 cultured pufferfish.

Species	*Takifugu obscurus*	*Takifugu rubripes*	*Takifugu bimaculatus*	*Takifugu flavidus*
Tetrodotoxin content (μg/g)	ND	ND	ND	ND
Toxicity level	D	D	D	D

Note: ND indicates “No detection”; D indicates “Non-toxic level”.

**Table 2:** Contents of general nutrients in the sperm of cultured *T. flavidus*. (Fresh weight g/100 g).

Items		Content	Items		Content
Nutrients (%)	Crude protein	17.47 ± 0.49 ^a^	Amino acids (g/100 g)	Lysine (Lys)	0.67 ± 0.01 ^c^
	Crude fat	1.71 ± 0.12 ^c^		∑EAA	2.34
	Crude ash	1.80 ± 0.06 ^b^		Aspartic acid (Asp **)	0.63 ± 0.01 ^b^
	Moisture	79.01 ± 0.45 ^c^		Serine(Ser)	0.34 ± 0.00 ^d^
Minerals (mg/kg)	Calcium (Ca)	42.63 ± 1.12 ^b^		Glutamic acid (Glu **)	0.90 ± 0.01 ^c^
	Potassium (K)	3336.67 ± 25.17 ^c^		Glycine (Gly **)	0.44 ± 0.00 ^c^
	Sodium (Na)	652.67 ± 4.04 ^d^		Alanine (Ala **)	0.41 ± 0.00 ^d^
	Magnesium (Mg)	150.09 ± 1.73 ^c^		Cysteine (Cys)	0.01 ± 0.00 ^b^
	Phosphorus (P)	2660.00 ± 32.06 ^b^		Tyrosine (Tyr)	0.15 ± 0.00 ^b^
	Zinc (Zn)	4.67 ± 0.21 ^d^		Histidine (His)	0.14 ± 0.00 ^c^
	Selenium (Se)	0.18 ± 0.06 ^d^		Arginine (Arg)	0.60 ± 0.01 ^d^
Amino Acids (g/100 g)	Threonine (Thr)	0.33 ± 0.00 ^c^		Proline (Pro)	0.26 ± 0.00 ^c^
	Valine (Val)	0.34 ± 0.00 ^c^		∑NEAA	3.88
	Methionine (Met)	0.09 ± 0.01 ^b^		∑TAA	6.21
	Isoleucine (Ile)	0.20 ± 0.01 ^c^		∑FAA	2.75
	Leucine (Leu)	0.49 ± 0.01 ^c^		∑EAA/∑TAA	37.57%
	Phenylalanine (Phe)	0.22 ± 0.01 ^c^			

Note: Lowercase letters indicate significant differences between different groups (*p* < 0.05). ** indicates umami amino acids; ∑EAA represents the total amount of essential amino acids; ∑NEAA represents the total amount of non-essential amino acids; ∑TAA represents the total amount of all amino acids; ∑FAA represents the total amount of umami amino acids.

**Table 3:** Evaluation of essential amino acids in the sperm of cultured *T. flavidus*.

Scoring Pattern	Thr	Val	Ile	Leu	Phe + Tyr	Met + Cys	Lys	Total	*EAAI*
*AAS*	0.47	0.39	0.29	0.40	0.35	0.15	0.70	2.76	0.27
CS	0.40	0.30	0.22	0.33	0.23	0.09	0.54	2.11	

**Table 4:** Sakaguchi reaction and antibacterial activity of protamine from *T. flavidus*.

	Sephadex G50 Gel Chromatography			CM Sepharose Fast Flow Ion Exchange Chromatography			Sephadex G25 Gel Chromatography	
	S-I	S-II	S-III	FF-I	FF-II	FF-III	G-I	G-II
Sakaguchi reaction	+++	−	−	−	+	+++	+++	−
Antibacterial activity	+++	−	−	−	−	++	+++	−

Note: − indicates no Sakaguchi reaction and no antibacterial activity detected; +, ++, and +++ represent the intensity of the Sakaguchi reaction and antibacterial activity, indicating weak, moderate, and strong, respectively.

**Table 5:** Amino acid composition of protamine from *T. flavidus*.

Amino Acid Species		Crude Protamine of Pufferfish (CPP)		Purified Protamine of Pufferfish (PPP)	
		Content (g/100 g)	Percentage (%)	Content (g/100 g)	Percentage (%)
Aspartic acid	Asp	2.10 ± 0.02	4.54 ± 0.04	1.78 ± 0.02	3.85 ± 0.05
Threonine	Thr	2.55 ± 0.02	5.52 ± 0.05	1.79 ± 0.01	3.86 ± 0.01
Serine	Ser	2.71 ± 0.02	5.87 ± 0.05	2.68 ± 0.01	5.79 ± 0.03
Glutamic acid	Glu	1.88 ± 0.02	4.07 ± 0.04	1.08 ± 0.02	2.33 ± 0.03
Glycine	Gly	3.80 ± 0.02	8.21 ± 0.04	2.99 ± 0.01	6.46 ± 0.02
Alanine	Ala	6.01 ± 0.04	13.00 ± 0.05	6.13 ± 0.04	13.25 ± 0.10
Cysteine	Cys	−	−	0.13 ± 0.02	0.29 ± 0.04
Viline	Val	2.80 ± 0.02	6.06 ± 0.03	2.42 ± 0.02	5.22 ± 0.03
Methionine	Met	0.54 ± 0.01	1.17 ± 0.01	0.17 ± 0.01	0.36 ± 0.03
Isoleucine	IIe	1.37 ± 0.01	2.95 ± 0.02	0.86 ± 0.01	1.85 ± 0.03
Leucine	Leu	2.65 ± 0.02	5.72 ± 0.03	1.73 ± 0.02	3.74 ± 0.04
Tyrosine	Tyr	0.99 ± 0.01	2.15 ± 0.02	0.27 ± 0.01	0.58 ± 0.01
Phenylalanine	Phe	0.84 ± 0.01	1.81 ± 0.01	1.11 ± 0.02	2.39 ± 0.05
Lysine	Lys *	6.36 ± 0.01	13.75 ± 0.02	12.5 ± 0.01	27.02 ± 0.19
Hlstidine	His *	0.14 ± 0.01	0.30 ± 0.02	0.71 ± 0.01	1.53 ± 0.02
Argnine	Arg *	9.18 ± 0.01	19.85 ± 0.03	17.0 ± 0.06	36.90 ± 0.17
Proline	Pro	2.32 ± 0.03	5.03 ± 0.06	1.53 ± 0.02	3.31 ± 0.05

Note: “−” indicates values below the detection limit (<0.002), not detected; “*” represents basic amino acids.

**Table 6:** Distribution proportion of the secondary structure of protamine.

Secondary Structure	A-Helix	Β-Sheet	Β-Turn	Random Coil
Percentage (%)	0.00	51.40	10.40	38.20

## Data Availability

The data presented in this study are available on request from the corresponding author.The data used to support the findings of this study can be made available by the corresponding author upon request.
